# A Hierarchical Model for Longitudinal and Intraparticle Diffusion Coefficients in Liquid Chromatography

**DOI:** 10.1021/acs.analchem.5c02838

**Published:** 2025-09-22

**Authors:** Alessandra Adrover, Gert Desmet

**Affiliations:** † Dipartimento di Ingegneria Chimica Materiali Ambiente, Sapienza Università di Roma, Rome 00184, Italy; ‡ Department of Chemical Engineering, 70493 Vrije Universiteit Brussel, Brussels 1050, Belgium

## Abstract

The model for the effective longitudinal diffusion coefficient *D*_eff_, recently derived from the two-zone moment analysis approach, is adapted to predict the intraparticle diffusion coefficient *D*_part_ of the general rate model for chromatography. The model for *D*_part_, coupled with the model for *D*_eff_, allows us to predict with great accuracy numerical data for *D*_eff_ obtained by solving the detailed diffusion-adsorption transport model, via the Brenner–Adler method of moments, in different 2D and 3D hierarchical retentive porous structures. The proposed models make explicit the dependence of *D*_eff_ and *D*_part_ on the obstruction factors of the macroporous and mesoporous zone, internal and external porosity, equilibrium constant, specific surface area, and surface diffusion coefficient. Detailed numerical simulations also allowed us to verify the inaccuracy of the parallel-connection model for *D*_part_ when surface diffusion in the stationary phase layer contributes significantly to the transport of the analyte in the mesoporous zone.

## Graphical abstract

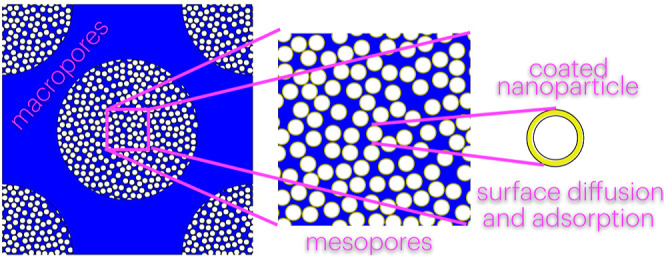

## Introduction

The peak-parking technique^1–4^ offers a way to experimentally measure the effective longitudinal diffusion coefficient *D*_eff_ and determine the B-term contribution^5,6^ which, in most columns, constitutes a significant fraction (order of 30–40%) of the plate height at the minimum of the van Deemter curve. This method has become a trusted tool in HPLC column characterization, helping to assess factors such as the structure of the stationary phase, surface chemistry, and retention mechanisms. *D*_eff_ values can also be examined for the determination of the intraparticle diffusivity *D*_part_ in the mesoporous region of the chromatographic bed. *D*_part_ is a crucial parameter in a column’s efficiency. It determines the B-term dispersion and the intraparticle mass transfer resistance and also contributes significantly to the eddy-dispersion.^7^ *D*_part_ in turn is influenced by the obstruction effect of the intraparticle mesopore space, the actual composition of the mobile phase along the coordinate perpendicular to the mesopore walls,^8,9^ and the diffusion rate experienced in (partition-based chromatography) or on the stationary phase (adsorption-based chromatography).^10^ In the past, several models have been proposed to describe this, some based on the sum of parallel diffusion fluxes,^1^ some based on the effective medium theory,^6^ and some including two-zone^11^ and even three- and four-zone models.^12^ *D*_part_ is a key parameter of the general rate (GR) model of chromatography^13,14^ which in the purely diffusive case (zero eluent velocity) reads as

1 $$\begin{array}{l} \begin{array}{l} \begin{array}{l} \frac{\partial c_{m}}{\partial t}=D_{m}\nabla^{2}c_{m},,x\in \Omega_{m}\\ \frac{\partial c_{part}}{\partial t}=D_{part}\nabla^{2}c_{part},\;x\in \Omega_{part} \end{array}\\ c_{part}{|}_{\partial \Omega_{m}}=K_{part}c_{m}{|}_{\partial \Omega_{m}} \end{array}\\ -D_{m}\nabla c_{m}\cdot n{|}_{\partial \Omega_{m}}=-D_{part}\nabla c_{part}\cdot n{|}_{\partial \Omega_{m}} \end{array}$$

where **n** is the unit norm vector at the fluid/particle interface ∂Ω_*m*_, *c*_*m*_ [mol/m^3^] and *c*_part_ [mol/m^3^] are analyte concentrations in the mobile zone Ω_*m*_ (volume *V*_Ω_*m*__) and in the stationary/particle zone Ω_part_ (total volume *V*_Ω_part__ including mesopores) shown in Figure 1A, *D*_*m*_ and *D*_part_ the diffusion coefficients in Ω_*m*_ and Ω_part_, and *K*_part_ the partition coefficient relating *c*_*m*_ and *c*_part_ at the boundary ∂Ω_*m*_

2 $$K_{part}=\varepsilon_{\mathrm{int}}\left(1+K_{eq}a\right)$$

and accounting for the mesoporous zone characteristic parameters, namely, the internal porosity $\varepsilon_{\mathrm{int}}=\frac{volume\;of\;the\;mesopores}{total\;particle\;volume}=\frac{V_{\Omega_{m,part}}}{V_{\Omega_{part}}}$ , the specific surface $a\left[m^{-1}\right]=\frac{surface\;of\;the\;mesopores}{volume\;of\;the\;mesopores}=\frac{S}{V_{\Omega_{m,part}}}$ , and the equilibrium constant $K_{eq}\left[m\right]=\frac{k_{a}\left[ms^{-1}\right]}{k_{d}\left[s^{-1}\right]}$ , i.e., the ratio between the adsorption *k*_*a*_ and desorption *k*_*b*_ rate constants.

**Figure 1:**
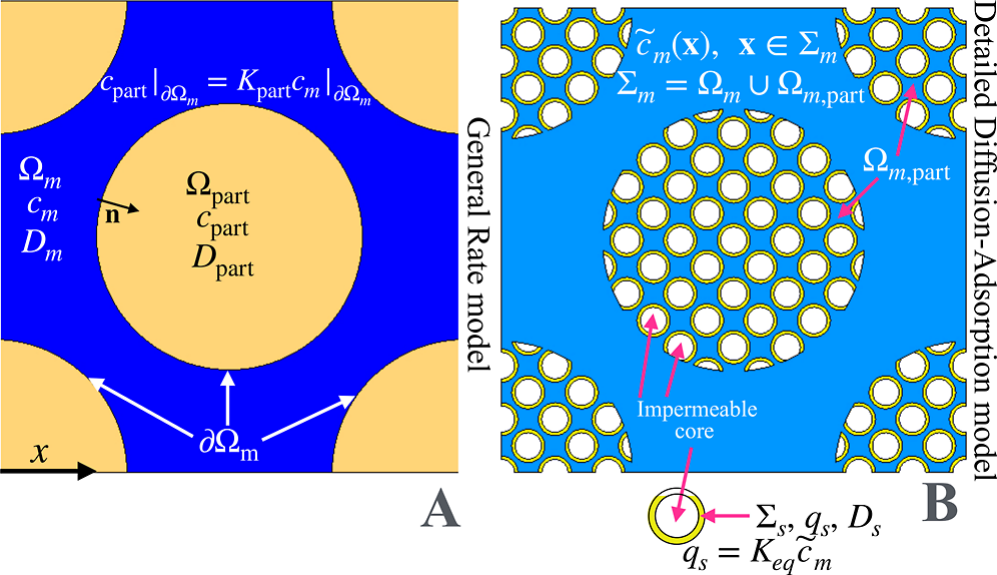
Domains, variables, and parameters entering the general rate model (A) and the detailed diffusion-adsorption model (B). The blue region in figure A indicates Ω_*m*_. The azure region in figure B indicates Σ_*m*_.

Being derived from a volume-averaging approach,^15^ the GR model considers the mesoporous region Ω_part_ as an isotropic and homogeneous solid phase, in which analyte transport occurs solely by diffusion with an effective diffusion coefficient *D*_part_ accounting for both hindered diffusion and surface adsorption in the mesopores, i.e., *D*_part_ = *f*(ε_int_,γ_*m*,int_^0^,*K*_*eq*_ *a*,*D*_*s*_), where γ_*m*,int_^0^ is the obstruction factor experienced by the analyte when diffusing in the particle mesopores Ω_*m*,part_ and *D*_*s*_ is the surface diffusion coefficient, i.e., the analyte diffusivity in the stationary phase layer covering the walls of the mesopores. When experimental data of *D*_eff_ are available, it is possible to follow a top-down approach, which consists in adopting a model for *D*_eff_, for example, Maxwell’s or Torquato’s model^6,16,17^ derived from the effective medium theory (EMT) or the model recently proposed by Adrover et al.^18,19^ based on the two-zone moment approach (TZMA).^20^ The model for *D*_eff_ can be used to estimate *D*_part_, as a best–fit parameter, for different column types and different retention conditions, thus investigating how *D*_part_ is affected by the mesopore geometry and physical parameters. When adopting a computational approach,^21–24^ a bottom-up strategy can be followed, which consists in solving a detailed transport model of the analyte in the macro–mesoporous chromatographic structure. In this way, it is possible to control the geometry of the mesoporous zone (ε_int_, γ_*m*,int_^0^), the retentive conditions (*K*_*eq*_ *a*), and the surface diffusion (*D*_*s*_),^25^ thus analyzing how all these mesoporous–scale parameters influence the intraparticle diffusivity *D*_part_, which, in turn, controls the macroscopic effective longitudinal diffusion coefficient *D*_eff_. Computationally, this is only possible if one adopts a method that allows estimation of *D*_eff_ by solving the transport equations of the detailed model only on a unit cell representative of the entire chromatography bed. In this paper, the Brenner–Adler method of moments^26^ is adopted to estimate *D*_eff_ from spatial moments of the analyte concentrations ${\mathrm{c̃}}_{m}$ and *q*_*s*_ satisfying the detailed diffusion-adsorption (DDA) model^26,27^ in the limiting case of negligible mass transfer resistance at the fluid/solid interface and infinitely fast adsorption/desorption process

3 $$\begin{array}{l} \frac{\partial {\mathrm{c̃}}_{m}}{\partial t}=D_{m}\nabla^{2}{\mathrm{c̃}}_{m},,x\in \Sigma_{m}=\Omega_{m}\cup \Omega_{m,part}\\ \frac{\partial q_{s}}{\partial t}=D_{s}\nabla_{s}^{2}q_{s}-D_{m}\nabla {\mathrm{c̃}}_{m}\cdot n{|}_{\Sigma_{s}},,x\in \Sigma_{s}\\ q_{s}{|}_{\Sigma_{s}}=K_{eq}{\mathrm{c̃}}_{m}{|}_{\Sigma_{s}} \end{array}$$

In this setting, ${\mathrm{c̃}}_{m}$ [mol/m^3^] is the analyte concentration in the mobile zone Σ_*m*_ which includes both macropores Ω_*m*_ and mesopores Ω_*m*,part_, thus extending over a total volume *V*_Σ_*m*__ = *V*_Ω_*m*__ + *V*_Ω_*m*,part__, where *V*_Ω_*m*,part__ is the fluid volume within the mesoporous particle (see Figure 1B). Correspondingly, *q*_*s*_ [mol/m^2^] is the analyte concentration in the stationary phase layer Σ_*s*_ (total surface area *S*) covering the walls of the mesopores (see Figure 1B), where the analyte is adsorbed and diffuses^28^ with diffusion coefficient *D*_*s*_ [Fn fn1]. By adopting the Brenner–Adler method of moments, we analyzed and quantified the influence of retentive conditions and surface diffusion on *D*_eff_ for different geometries and internal porosities of the mesoporous zone. This allowed us to verify the reliability and accuracy of a model for *D*_part_, which was used in conjunction with a previously developed model for *D*_eff_, which is able to predict the numerical data of *D*_eff_ for the different geometries investigated and throughout the wide range of values of *K*_*eq*_ *a* and *D*_*s*_ analyzed. The approach here proposed to estimate *D*_part_ and *D*_eff_ is not as accurate and detailed as that recently implemented by Tallarek et al.^29^ combining molecular dynamics simulations in a single-mesopore model with Brownian dynamics simulations in hierarchical porosity models based on physical reconstructions of monolithic silica columns. However, the estimate of *D*_eff_ via the Brenner–Adler moment method in relatively simple hierarchical macropore–mesopore models permits us to assess the validity of a general model for *D*_part_ that overcomes the intrinsic limitations of the following oversimplified model

4 $$\frac{D_{part}^{∥}}{D_{m}}=\frac{\gamma_{m,\mathrm{int}}^{0}}{1+K_{eq}a}+\frac{K_{eq}a}{1+K_{eq}a}\frac{D_{s}}{D_{m}}$$

derived from the parallel-connection assumption that surface and pore diffusion occur in parallel.^6,30^

The parallel-connection model, eq 4, is based on the idea of representing the mesoporous zone as a bundle of cylindrical mesopores arranged in parallel, although with a certain degree of tortuosity. However, a more realistic depiction of the mesoporous zone is as a sintered network of nanoscale spheres—a ternary system composed of the mobile phase liquid occupying the mesopores, impermeable silica particles, and a stationary phase layer coating the silica surface. This latter is the idea of the mesoporous zone that inspired the 2D and 3D model geometries investigated.

## Materials and Methods

According to the homogenization method proposed by Brenner and Alder,^26^ the effective longitudinal diffusion coefficient *D*_eff_ of an analyte satisfying the transport eq 3 of the DDA model can be evaluated as

5 $$D_{eff}=D_{m}m_{\Sigma_{m}}\left(1-{\left\langle \nabla b_{m}\cdot e_{x}\right\rangle }_{\Sigma_{m}}\right)+D_{s}m_{\Sigma_{s}}\left(1-{\left\langle \nabla_{s}b_{s}\cdot e_{x}\right\rangle }_{\Sigma_{s}}\right)$$

where *m*_Σ_*m*__ and *m*_Σ_*s*__ = 1 – *m*_Σ_*m*__ are the fractions of analyte particles in the mobile zone Σ_*m*_ and stationary phase layer Σ_*s*_, respectively

6 $$\begin{array}{l} m_{\Sigma_{m}}={\left(1+\frac{K_{eq}S}{V_{\Sigma_{m}}}\right)}^{-1}={\left(1+K_{eq}a\frac{V_{\Omega_{m,part}}}{V_{\Sigma_{m}}}\right)}^{-1}\\ {=\left(1+K_{eq}a\frac{\left(1-\varepsilon_{e}\right)\varepsilon_{\mathrm{int}}}{\varepsilon_{tot}}\right)}^{-1} \end{array}$$

ε_*e*_ = *V*_Ω_*m*__/(*V*_Ω_*m*__ + *V*_Ω_part__) is the external porosity of the bed, ε_tot_ = *V*_Σ_*m*__/(*V*_Ω_*m*__ + *V*_Ω_part__) = ε_*e*_ + (1 – ε_*e*_)­ε_int_ is the total porosity of the bed, and *b*_*m*_ [m] and *b*_*s*_ [m] are the b-fields satisfying the following stationary transport equations and boundary conditions on the unit cell Σ = Σ_*m*_ ∪ Σ_*s*_

7 $$\begin{array}{l} D_{m}\nabla^{2}b_{m}=0,,x\in \Sigma_{m}\\ K_{eq}D_{s}\nabla_{s}^{2}b_{s}=-D_{m}\left(\nabla b_{m}-e_{x}\right)\cdot n{|}_{\Sigma_{s}}\\ b_{m}{|}_{\Sigma_{s}}=b_{s}{|}_{\Sigma_{s}} \end{array}$$

plus periodic boundary conditions at the edges of the unit cell Σ. The vector **e**_*x*_ in eq 7 is the unit vector along the longitudinal direction *x*.

Figure 2A–C shows the 2D unit cells Σ (ε_*e*_ = 0.5) characterized by three different geometries of the mesoporous zone investigated, namely, a square array of circles (geometry A), an array of circles and ellipses (geometry B), and a random arrangement of circles (geometry C). Figure 3B shows the 3D unit cell of geometry D (simple cubic array of spheres, ε_*e*_ ≃ 0.49) and the geometry of the mesoporous zone made by 100 equal spheres packed in a sphere^31^ (ε_int_ ≃ 0.48). The coupled transport eq 7 for the b-fields has been solved by Finite Element Approach (FEM) in Comsol Multiphysics 6.3. The coefficient form partial differential equation (PDE) physics coupled with the coefficient form boundary PDE physics have been utilized with Lagrangian quadratic shape functions. The MUltifrontal Massively Parallel Sparse direct Solver (MUMPS) linear solver was employed with a relative tolerance of 1 × 10^–6^. The triangular mesh of 2D geometries (Figure 3A) consists of approximately 7 × 10^5^ domain elements, 1 × 10^5^ boundary elements (see Figure 3A). The tetrahedral mesh of the 3D geometry (Figure 3B) consists of approximately 3 × 10^6^ domain elements, 4 × 10^5^ boundary elements, and 3 × 10^4^ edge elements (see Figure 3B). Results are verified to be mesh independent in the whole range of *K*_*eq*_ *a* and *D*_*s*_/*D*_*m*_ values investigated.

**Figure 2:**
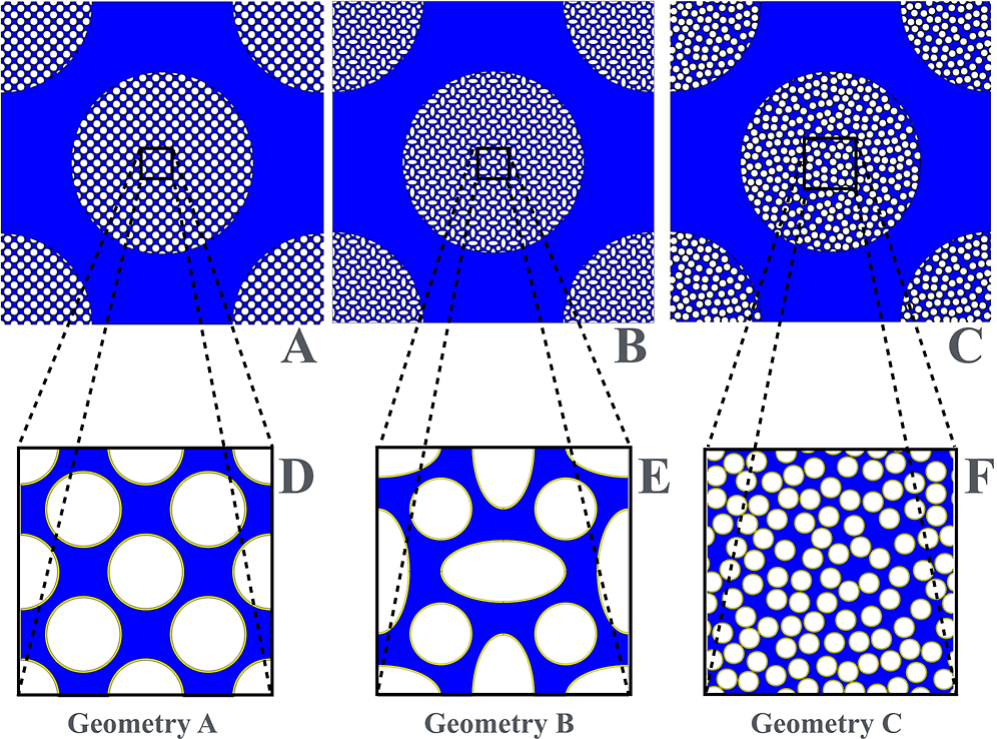
2D unit cells with different geometries of the mesoporous zone. (A) 2D array of circles; (B) 2D array of circles and ellipses; and (C) 2D random arrangement of circles. The three insets (D–F) show the periodic unit cells of the mesoporous zone.

**Figure 3:**
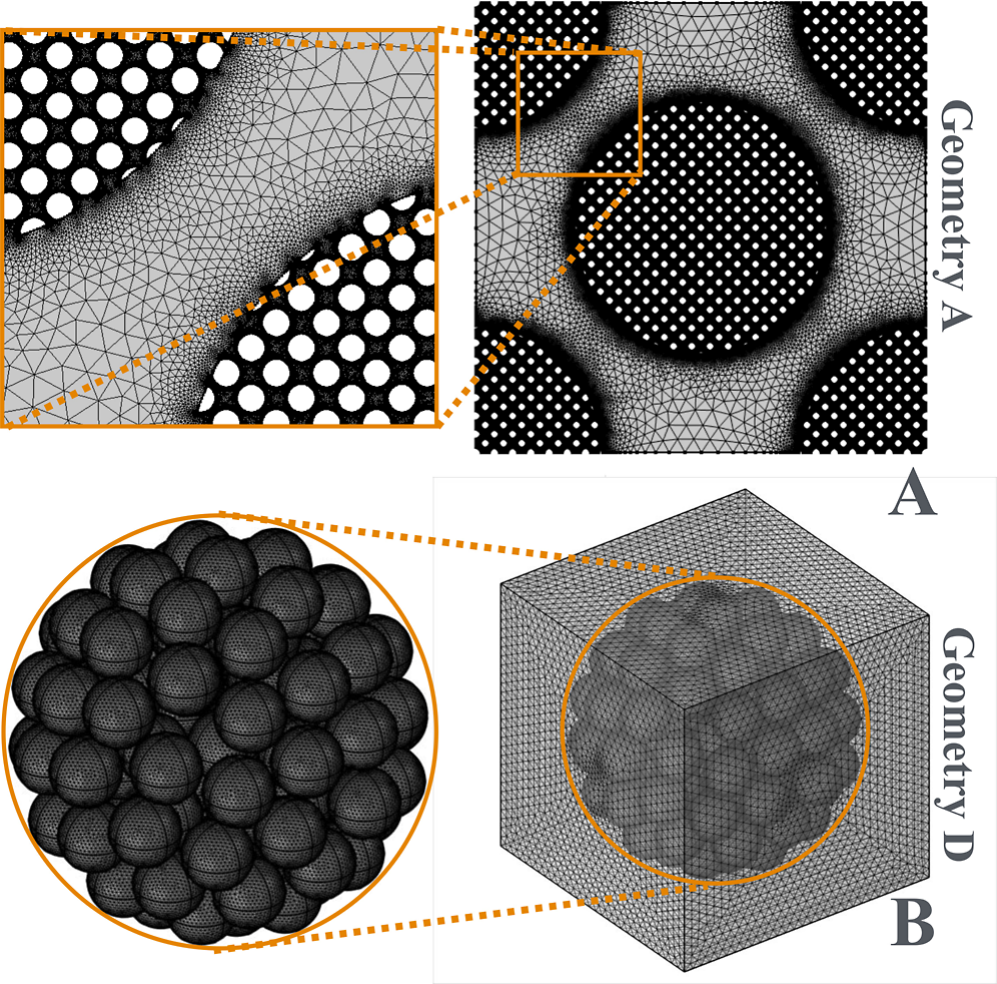
Mesh adopted for the 2D and 3D periodic unit cells. (A) 2D geometry A and (B) 3D geometry D.

## Results and Discussion

Numerical results for the rescaled effective longitudinal diffusion coefficient γ_eff_ = *D*_eff_/*D*_*m*_ vs *K*_*eq*_ *a* for different values of the rescaled surface diffusion coefficient *D*_*s*_/*D*_*m*_ ∈ [0, 0.5] are shown in Figure 4 for geometry A with ε_*e*_ = 0.5 and ε_int_ = 0.4, corresponding to a total porosity of ε_tot_ = 0.7. It can be observed that all the curves satisfy the limiting behavior $\lim_{K_{eq}a\to 0}\gamma_{eff}=\gamma_{\Sigma_{m}}^{0}$ , where $\gamma_{\Sigma_{m}}^{0}$ is the obstruction factor of Σ_*m*_. Moreover, $\lim_{K_{eq}a\to \infty }\gamma_{eff}=0$ because the mesoporous zone is made by isolated particles, and the chromatographic bed is not a bicontinuous structure. The higher the surface diffusivity *D*_*s*_, the higher γ_eff_. For very low values of *D*_*s*_/*D*_*m*_, namely, *D*_*s*_/*D*_*m*_ ≤ 10^–2^, curves deviate only for very high values of *K*_*eq*_ *a* from the limit curve with zero surface diffusion

8 $$\gamma_{eff}{|}_{D_{s}=0}=m_{\Sigma_{m}}\gamma_{\Sigma_{m}}^{0}$$

while they are extremely sensitive to *D*_*s*_ for *D*_*s*_/*D*_*m*_ > 0.1 in a wide range of *K*_*eq*_ *a* values. γ_eff_ is rather insensitive to the geometry of the mesoporous phase, as shown in Figure 5A,B where the curves γ_eff_ vs *K*_*eq*_ *a* are shown for geometry A in comparison with geometry B (Figure 5A) and geometry C (Figure 5B) for the same external porosity ε_*e*_ = 0.5 and the same internal porosity ε_int_ = 0.4. Small differences between geometry A and geometry C can be observed for very low *K*_*eq*_ *a* values because the obstruction factor $\gamma_{\Sigma_{s}}^{0}$ for random circles (geometry C) is appreciably smaller that that for the ordered array of circles (geometry A). On the contrary, γ_eff_ is significantly affected by the internal porosity ε_int_ because this latter strongly influences the obstruction factor $\gamma_{\Sigma_{m}}^{0}$ and the behavior of the γ_eff_ vs *K*_*eq*_ *a* for *D*_*s*_/*D*_*m*_ ≥ 0.1, as shown in Figure 6, for geometry A and for two different internal porosities, namely, ε_int_ = 0.3, 0.4.

**Figure 4:**
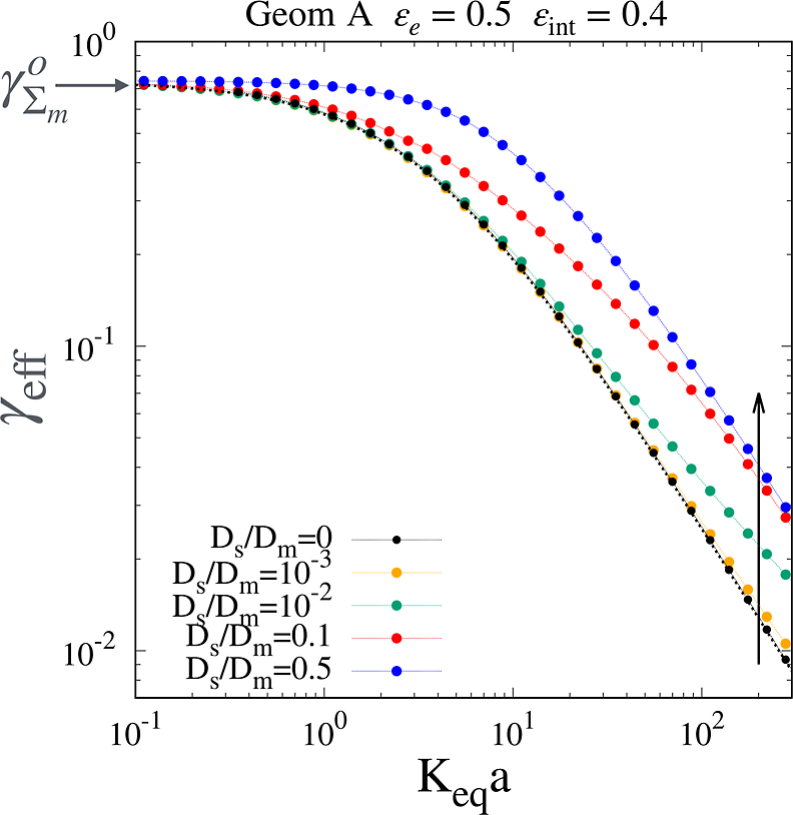
Numerical results for γ_eff_ vs *K*_*eq*_ *a* for geometry A with ε_*e*_ = 0.5 and ε_int_ = 0.4. The arrow indicates increasing values of *D*_*s*_/*D*_*m*_. The dotted black line shows eq 8 with $\gamma_{\Sigma_{m}}^{0}=0.752$ .

**Figure 5:**
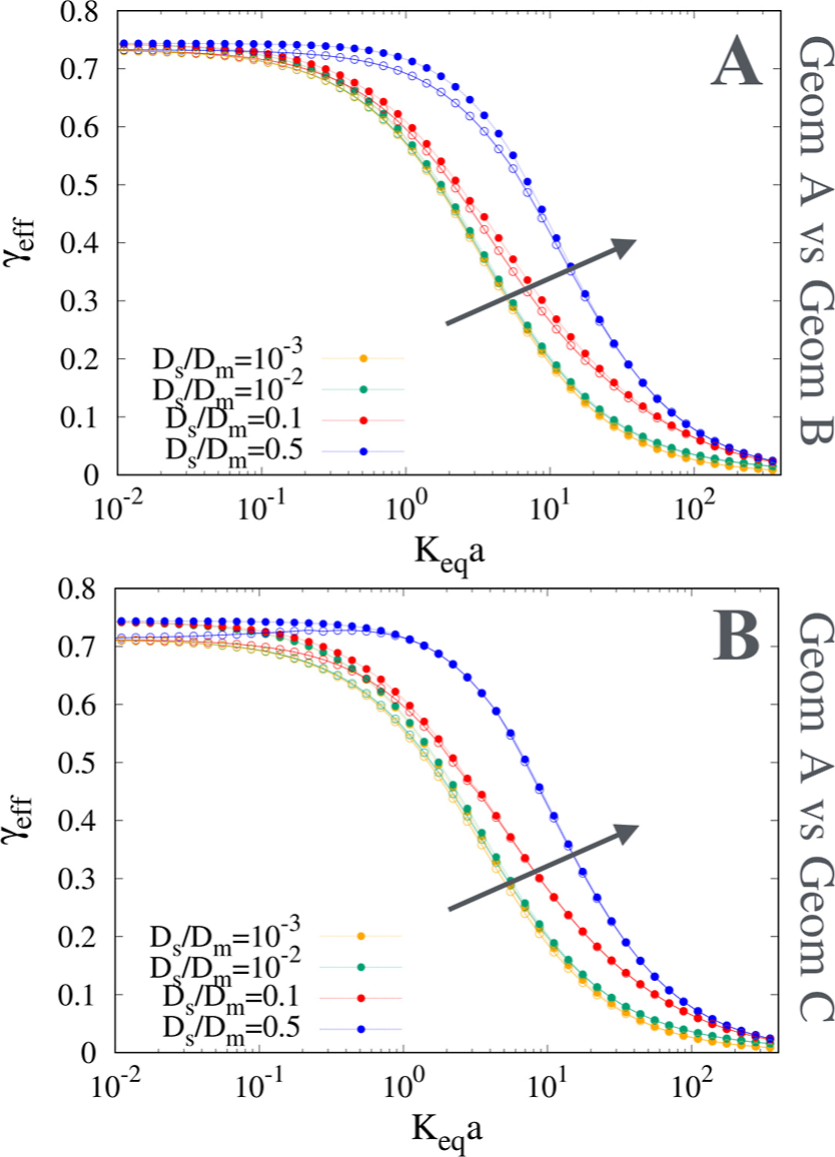
γ_eff_ vs *K*_*eq*_ *a* for ε_*e*_ = 0.5, ε_int_ = 0.4 and for different values of *D*_*s*_/*D*_*m*_. (A) Geometry A (full points) vs geometry B (open circles). (B) Geometry A (full points) vs geometry C (open circles). Arrows indicate increasing values of *D*_*s*_/*D*_*m*_.

**Figure 6:**
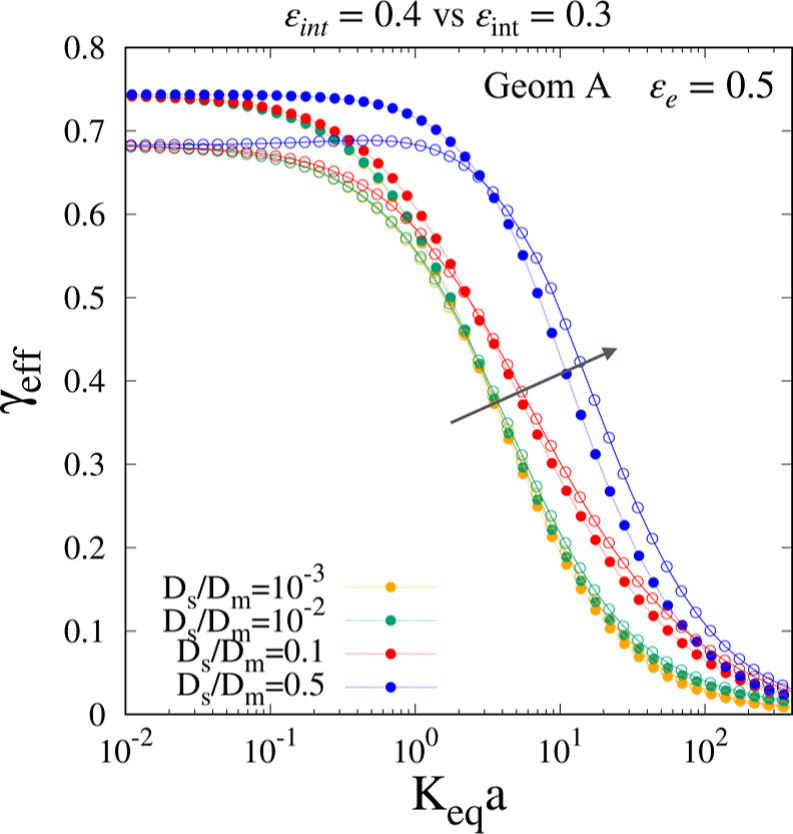
γ_eff_ vs *K*_*eq*_ *a* for ε_*e*_ = 0.5 for geometry A with ε_int_ = 0.4 (full points) and ε_int_ = 0.3 (open circles). The arrow indicates increasing values of *D*_*s*_/*D*_*m*_.

### Modeling *D*_eff_ and *D*_part_

Recently, a general model for *D*_eff_, derived from the TZMA approach, has been proposed as a valid alternative to the EMT models used so far in the literature. Its advantage is that it does not require any fitting parameter because all the necessary information is embedded into the experimentally accessible geometric obstruction factor $\gamma_{\Omega_{m}}^{0}$ of the mobile phase Ω_*m*_, originating from the tortuosity of the through-pore space (limiting case of fully solid particles without any retention). In its more general version, reliable for diluted and concentrated packings, the model reads as

9 $$\gamma_{eff}=\frac{\varepsilon_{e}\gamma_{\Omega_{m}}^{0}+\left(1-\varepsilon_{e}\gamma_{\Omega_{m}}^{0}+\beta -\delta \right)\alpha_{part}+\delta \alpha_{part}^{1-\varepsilon_{e}}}{\varepsilon_{e}\left(1+k^{\prime \prime }\right)\left(1+\left(\beta -\delta \right)\alpha_{part}+\delta \alpha_{part}^{1-\varepsilon_{e}}\right)}$$

where α_part_ is the relative particle permeability^6^ defined with respect to the zone retention factor *k*″

10 $$\begin{array}{l} \alpha_{part}=k^{\prime \prime }\frac{\varepsilon_{e}}{\left(1-\varepsilon_{e}\right)}\frac{D_{part}}{D_{m}}\\ k^{\prime \prime }=K_{part}\frac{V_{\Omega_{part}}}{V_{\Omega_{m}}}=\varepsilon_{\mathrm{int}}\left(1+K_{eq}a\right)\frac{1-\varepsilon_{e}}{\varepsilon_{e}} \end{array}$$

and β and δ are geometric parameters that depend solely on ε_*e*_ and $\gamma_{\Omega_{m}}^{0}$ as follows

11 $$\beta =\frac{\varepsilon_{e}\left(1-\gamma_{\Omega_{m}}^{0}\right)}{\left(1-\varepsilon_{e}\right)},,\delta =\frac{\left(\frac{n}{n+1-\varepsilon_{e}}-\gamma_{\Omega_{m}}^{0}\right)}{\left(1-\varepsilon_{e}\gamma_{\Omega_{m}}^{0}\right)}$$

where *n* = 1, 2 for cylindrical and spherical particles, respectively. In the diluted case (ε_*e*_ ≥ 0.5), the model attains the simplified form

12 $$\gamma_{eff}=\frac{\varepsilon_{e}\gamma_{\Omega_{m}}^{0}+\left(1-\varepsilon_{e}\gamma_{\Omega_{m}}^{0}+\beta \right)\alpha_{part}}{\varepsilon_{e}\left(1+k^{\prime \prime }\right)\left(1+\beta \alpha_{part}\right)}$$

because $\gamma_{\Omega_{m}}^{0}≃\frac{n}{n+1-\varepsilon_{e}}$ (Maxwell obstruction factor for diluted suspensions), that implies δ = 0.

The model applies to both fully porous and shell-porous particles by expressing *D*_part_ and *k*″ as

13 $$D_{part}=D_{pz}\left(\frac{n}{n+\rho^{n+1}}\right),,{,k}^{\prime \prime }=K_{pz}\frac{V_{\Omega_{pz}}}{V_{\Omega_{m}}}$$

where ρ = *d*_core_/*d*_part_ is the relative diameter of the solid particle core. *D*_pz_ and *K*_pz_ are the diffusion coefficient and the partition coefficient in the mesoporous zone Ω_pz_ that can be the entire particle if ρ = 0 or only the shell layer for ρ > 0. In the case of fully porous particles, Ω_part_ = Ω_pz_, *D*_part_ = *D*_pz_, and *K*_part_ = *K*_pz_. To compute *D*_pz_ needed in eq 13, we should zoom in on the mesopore level. However, at this level, we again see a ternary medium (mobile phase liquid occupying the mesopores and impermeable nanoparticles coated with a stationary phase layer cf. Figure 2) that should obey the same phenomenological laws for the effective diffusion as described by eqs 9–13 for the whole-column level (mobile phase liquid occupying the macropores of a packing of shell-porous particles). Assuming the mesopore space consists of a packing of surface-coated nanoparticles (cf. Figure 2) with surface diffusion *D*_*s*_, the translation of eqs 9–13 to the mesopore space requires that (i) γ_eff_ in eq 9 is replaced by *D*_pz_/*D*_*m*_. (ii) The mobile phase Ω_*m*_ is replaced by the mobile phase internal to the mesoporous particle Ω_*m*,part_, thus replacing ε_*e*_ with ε_int_ and $\gamma_{\Omega_{m}}^{0}$ with γ_*m*,int_^0^. (iii) The zone retention factor *k*″ is replaced with *K*_*eq*_ *a* because *K*_pz_ must be replaced by *K*_*eq*_ and *V*_Ω_pz__/*V*_Ω_*m*__ with *a* = *S*/*V*_Ω_*m*,part__. (iv) *D*_part_ is replaced by $\frac{D_{s}}{D_{m}}\frac{n}{n+1}$ by adopting the Hashin–Shtrikman formulation^6^ in eq 13 with the assumption that the coating (with internal diffusion *D*_*s*_) on the nanoparticles is so thin that we can put ρ = 1 in eq 13.

The resulting expression of *D*_pz_/*D*_*m*_ reads as

14 $$\frac{D_{pz}}{D_{m}}=\frac{\varepsilon_{\mathrm{int}}\gamma_{m,\mathrm{int}}^{0}+\left(1-\varepsilon_{\mathrm{int}}\gamma_{m,\mathrm{int}}^{0}+\beta_{\mathrm{int}}-\delta_{\mathrm{int}}\right)\alpha_{\mathrm{int}}+\delta_{\mathrm{int}}\alpha_{\mathrm{int}}^{1-\varepsilon_{\mathrm{int}}}}{\varepsilon_{\mathrm{int}}\left(1+K_{eq}a\right)\left(1+\left(\beta_{\mathrm{int}}-\delta_{\mathrm{int}}\right)\alpha_{\mathrm{int}}+\delta \alpha_{\mathrm{int}}^{1-\varepsilon_{\mathrm{int}}}\right)}$$

15 $$\beta_{\mathrm{int}}=\frac{\varepsilon_{\mathrm{int}}\left(1-\gamma_{m,\mathrm{int}}^{0}\right)}{\left(1-\varepsilon_{\mathrm{int}}\right)},,\delta_{\mathrm{int}}=\frac{\left(\frac{n}{n+1-\varepsilon_{\mathrm{int}}}-\gamma_{m,\mathrm{int}}^{0}\right)}{\left(1-\varepsilon_{\mathrm{int}}\gamma_{m,\mathrm{int}}^{0}\right)}$$

16 $$\alpha_{\mathrm{int}}=K_{eq}a\frac{\varepsilon_{\mathrm{int}}}{1-\varepsilon_{\mathrm{int}}}\frac{D_{s}}{D_{m}}\frac{n}{n+1}$$

### Comparison with Numerical Results

Figure 7A,B shows the excellent agreement between model predictions based on eqs 14–16 and numerical results for *D*_pz_/*D*_*m*_ vs *K*_*eq*_ *a* obtained by solving the b-field equations, eq 7, within different 2D and 3D periodic unit cells representative of the mesoporous zone, namely, the 2-d array of circles (Geom A, Figure 2D), the 2D random arrangement of circles (Geom C, Figure 2F), the 3D face-centered cubic array of spheres (fcc), and a 3D close random packing (CRP) of 100 equal spheres (ε_int_ = 0.375) with periodic boundary conditions generated using a collision-driven packing algorithm.^32^ The values of the mesopore obstruction factor γ_*m*,int_^0^, for the different mesopore unit cells investigated, are reported in Table 1. Indeed, γ_*m*,int_^0^ is the unique parameter entering the *D*_pz_ model, eq 14, and is obtained by solving the *b*_*m*_-field equation in the mesopores Ω_*m*,part_ in the impermeable case, i.e., in the limiting case of no superficial adsorption, *K*_*eq*_ *a* = 0. Figure 7A,B also shows that the parallel-connection model, eq 4, significantly overestimates *D*_pz_ especially for intermediate/large values of *D*_*s*_/*D*_*m*_.

**Figure 7:**
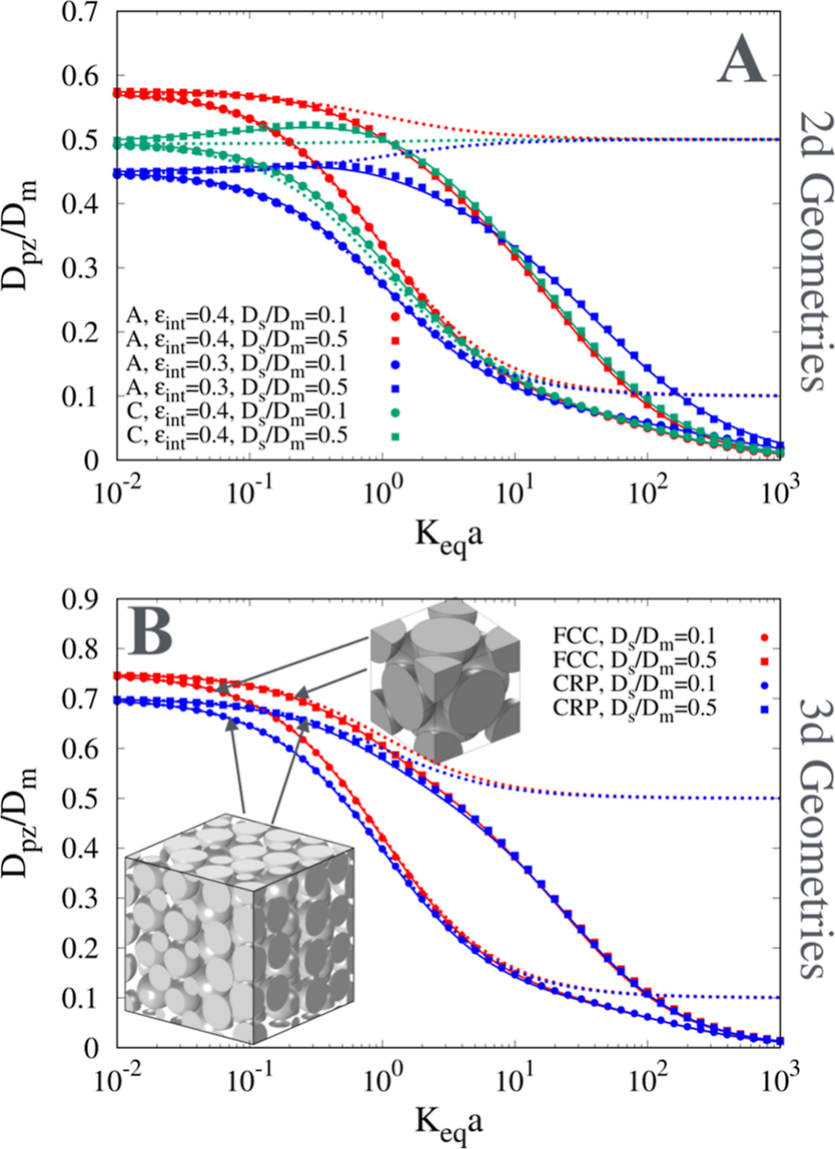
Comparison between numerical results (points) and model predictions for *D*_pz_/*D*_*m*_ vs *K*_*eq*_ *a* for *D*_*s*_/*D*_*m*_ = 0.1, 0.5. Continuous lines: TZMA model, eq 14. Dashed lines: parallel-connection model, eq 4. (A) 2D geometry A (ε_int_ = 0.3, 0.4) and geometry C (ε_int_ = 0.4). (B) 3D geometries: fcc array of spheres (fcc, ε_int_ = 0.4) and a close random packing of 100 equal spheres (CRP, ε_int_ = 0.375).

**Table 1:** γ_*m*,int_^0^ for Different Mesoporous Geometries^a^

	geom A	geom B	geom C
γ_*m*,int_^0^	0.575 [0.4]	0.545 [0.4]	0.505 [0.4]

	geom A	fcc	CRP
γ_*m*,int_^0^	0.448 [0.3]	0.750 [0.4]	0.703 [0.375]

a The corresponding porosity ε_int_ is indicated in square brackets [·].

By replacing eq 14 for *D*_pz_(ε_int_,γ_*m*,int_^0^,*K*_*eq*_ *a*,*D*_*s*_) into eqs 9–13 for $\gamma_{eff}=f\left(\varepsilon_{e},\gamma_{\Omega_{m}}^{0},K_{eq}a,D_{part}\right)$ , we obtain a complete model for γ_eff_ that makes explicit the dependence on the internal and external porosities, the equilibrium constant, the specific surface area, and surface diffusion coefficient. The dependence on the specific geometries of the macropore and mesopore networks is embedded in the obstruction factors $\gamma_{\Omega_{m}}^{0}$ and γ_*m*,int_^0^. For all the 2D geometries investigated, the macropore structure is the same (square array of circles, ε_*e*_ = 0.5) and the corresponding macropore obstruction factor is $\gamma_{\Omega_{m}}^{0}=0.652$ . For the 3D geometry D, the macropore structure is a simple cubic array of spheres (ε_*e*_ = 0.49), and the corresponding obstruction factor is $\gamma_{\Omega_{m}}^{0}=0.789≃2/\left(3-\varepsilon_{e}\right)$ . Regarding the obstruction factor γ_*m*,int_^0^ in the mesoporous zone (ε_int_ ≃ 0.48), since the 3D geometry D does not allow us to identify a periodic cell, and thus to estimate γ_*m*,int_^0^, we assumed $\gamma_{m,\mathrm{int}}^{0}=\gamma_{\Omega_{m}}^{0}$ as the internal and external porosities are very similar and in the range of validity of the Maxwell equation. For all the geometries, *D*_part_ = *D*_pz_ because only fully porous particles are considered (cf. Figures 2 and 3). Figure 8A shows the comparison, for geometry A, between numerical results for γ_eff_ vs *K*_*eq*_ *a* (bottom *x* axis) and *k*″ (top *x* axis) and the predictions of the model for γ_eff_, eq 9 with *D*_pz_ = *D*_part_ estimated from the parallel-connection model, eq 4. It can be observed that the adoption of *D*_part_^∥^ results in a significant overestimate of γ_eff_ for *D*_*s*_/*D*_*m*_ ≥ 0.1 over the entire range of zone retention factor *k*″ of practical interest, *k*″ ∈ [1, 30]. In contrast, the adoption of the TZMA model for *D*_pz_, eq 14, into the TZMA model for γ_eff_, eq 9, leads to a very accurate prediction of numerical data for γ_eff_ in the whole range of *D*_*s*_ and *k*″ values investigated, as shown in Figure 8B for the 2D geometry A, and in Figure 9A–C for the 2D geometries B and C and for the 3D geometry D.

**Figure 8:**
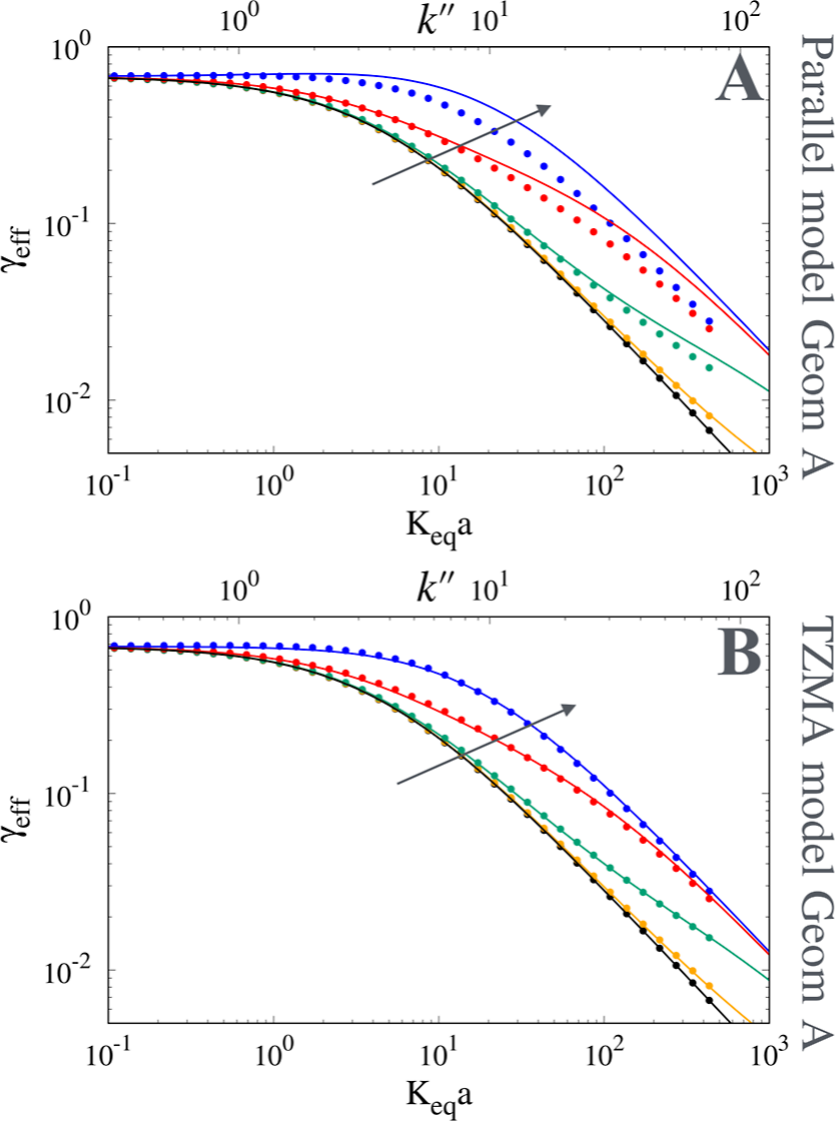
Comparison between numerical results (points) and model predictions (continuous lines) for γ_eff_ vs *K*_*eq*_ *a* (bottom *x* axis) and *k*″ (top *x* axis) for geometry A, ε_*e*_ = 0.5, ε_int_ = 0.3. (A) Parallel-connection model, eq 4. (B) TZMA model, eq 14. The arrow indicates increasing values of *D*_*s*_/*D*_*m*_ = 0, 10^–3^, 10^–2^, 0.1, and 0.5.

**Figure 9:**
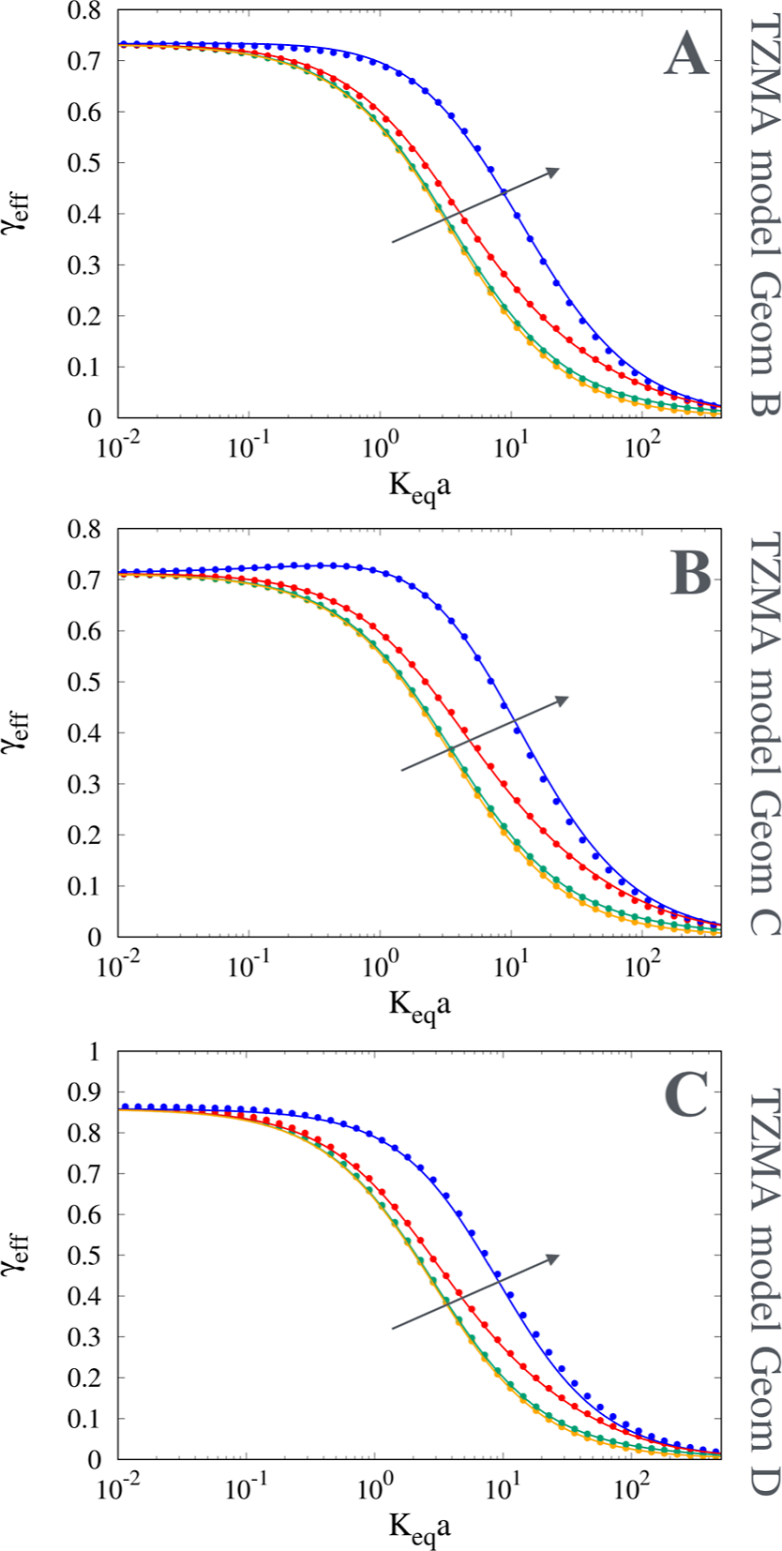
Comparison between numerical results (points) and model predictions (continuous lines) for γ_eff_ vs *K*_*eq*_ *a*. (A) 2D geometry B. (B) 2D geometry C. (C) 3D geometry D, ε_*e*_ ≃ 0.49, ε_int_ ≃ 0.48. The arrow indicates increasing values of *D*_*s*_/*D*_*m*_ = 10^–3^, 10^–2^, 0.1, and 0.5.

## Conclusions

The model proposed for *D*_eff_, eq 9, and *D*_pz_, eq 14, allows us to predict with great accuracy (error below 2%) numerical data for 2D and 3D hierarchical porous media in the whole range of *D*_*s*_/*D*_*m*_ and *K*_eq_ *a* values investigated. The 2D geometries analyzed resemble closely those of pillar array columns with fully porous pillars. The mesoporous structures of the 3D geometries are reasonable models of monodisperse silica-based materials. Numerical results for *D*_pz_ and *D*_eff_ show low sensitivity to the specific geometry of the mesoporous zone and high sensitivity to the internal porosity. We therefore believe that, even though the geometries analyzed are simplified models of the complex structure of the macropore/mesoporous zone, the models proposed for *D*_eff_ and *D*_pz_ can also be successfully applied to more realistic geometries^29^ that can be obtained via porous-media reconstructions by scanning transmission electron microscopy (mesopore space)^33^ and confocal laser scanning microscopy (macropore space).^34^ The application of the hierarchical model *D*_pz_–*D*_eff_ requires the preliminary estimate of the two obstruction factors $\gamma_{\Omega_{m}}^{0}$ and γ_*m*,int_^0^. While $\gamma_{\Omega_{m}}^{0}$ can be experimentally estimated, γ_*m*,int_^0^ must be obtained by a best fit of experimental data. A good first attempt value of γ_*m*,int_^0^ is represented by the Maxwell diluted limit value γ_*m*,int_^0^ ≃ *n*/(*n* + 1-ε_int_). Verification of the model is planned through application to a data set of experimental *D*_eff_ values obtained by Deridder et al.^35^ using peak parking on various first- and second-generation monolithic silica columns. The hierarchical model proposed in this paper is derived from the model for *D*_eff_ stemming from the TZMA approach. By adopting a similar strategy, different hierarchical models can also be constructed from other models of *D*_eff_ that can be found in the literature, such as the Torquato’s model^18^ stemming from EMT, as already proposed by Desmet and Deridder back in 2011.^6^

## Footnotes

a The operators ∇_*s*_ and ∇_*s*_^2^ = ∇_*s*_·(∇_*s*_) are the surface gradient and Laplace–Beltrami operators, respectively. Specifically, ∇_*s*_ = **I**_*s*_·∇ wherein **I**_*s*_ = **I** – **nn** is the surface idemfactor dyadic, **I** the idemfactor dyadic, and **n** a unit normal vector on Σ_*s*_.
